# Dysbiosis in Children With Neurological Impairment and Long-Term Enteral Nutrition

**DOI:** 10.3389/fnut.2022.895046

**Published:** 2022-06-22

**Authors:** Simona Panelli, Valeria Calcaterra, Elvira Verduci, Francesco Comandatore, Gloria Pelizzo, Elisa Borghi, Claudio Bandi, Gianvincenzo Zuccotti

**Affiliations:** ^1^Pediatric Clinical Research Center “Invernizzi”, Department of Biomedical and Clinical Sciences “L. Sacco”, University of Milan, Milan, Italy; ^2^Pediatric Department, “Vittore Buzzi” Children's Hospital, Milan, Italy; ^3^Pediatric and Adolescent Unit, Department of Internal Medicine, University of Pavia, Pavia, Italy; ^4^Department of Health Sciences, University of Milano, Milan, Italy; ^5^Pediatric Clinical Research Center “Invernizzi”, Department of Biosciences, University of Milan, Milan, Italy; ^6^Pediatric Surgery Department, “Vittore Buzzi” Children's Hospital, Milan, Italy

**Keywords:** gut microbiome, children, neurological impairment, dysbiosis, enteral nutrition

## Abstract

Severe neurological impairment (NI) is often accompanied by the need for artificial nutritional support, normally provided enterally (enteral nutrition [EN]) to ensure growth, counteract morbidity and mortality, and improve quality of life. On the other hand, long-term EN (LTEN) may contribute to the establishment, or exacerbation, of gastrointestinal disorders that may lead to malnutrition, which in turn is associated with alterations in gut microbiota (GM) composition and functional capacities. To the best of our knowledge, we investigated, for the first time in this study, the consequences of LTEN in a pediatric population in this pathological context. Using amplicon sequencing, we compared the fecal microbiota of a pediatric population suffering from severe NI and under LTEN to that of sex- and age-matched controls. The two groups presented evident differences in GM composition and a consistent differential clustering. In general, the taxonomic picture in NI children under LTEN seemed to mirror a profound dysbiotic condition, in which anti-inflammatory taxa appear severely depleted (among others, the *Clostridiales* families of *Lachnospiraceae* and *Ruminococcaceae*, and, within the latter, *Faecalibacterium* spp. and *Gemmiger* spp.), while known pathobionts (*Gammaproteobacteria* and *Klebsiella*) or emerging pathogens (e.g., *Synergistales, Cloacibacillus*, and *Fusobacterium*) were significantly enriched. Our data suggest that LTEN has a significant impact on the GM taxonomic composition in NI children. Even if other factors are probably at work, such as the bidirectional interaction between gastrointestinal impairment/immaturity and the central nervous system (CNS), the assumption of drugs, and physical inactivity, these data define possible routes and targets to try to alleviate this dysbiosis, with a view to better management of these patients and an improvement in their quality of life.

## Introduction

The gut microbiota (GM) is a complex ecological community that influences host physiology and susceptibility to disease through its metabolic activities and interactions with many systems and functions of the host (1). The human microbiota is estimated to contain 10^13^-10^14^ microbial cells, with a 1:1 microbial to human cells ratio (2), and is located predominantly in the large intestine of the gastrointestinal (GI) tract (3).

The microbiota normally establishes a mutually beneficial cohabitation with the host: it protects against pathogens, extracts nutrients and energy from the diet, and contributes to the maintenance of the immune response (4). Alterations of the GM, caused either by environmental or host-related factors, can impair its ability to maintain good health and lead to dysbiosis. Dysbiosis, defined as an “imbalance” between GM and host, is characterized by a reduction in microbial diversity and/or an increase in proinflammatory species (5) and has been associated with a plethora of gastro-intestinal-extra-intestinal disorders, such as inflammatory bowel diseases (IBDs), obesity, cancer, malnutrition, and neurological disorders (5).

Several evidence have reported a bidirectional interaction between the GI tract and the central nervous system (CNS). Communication occurs through the autonomic nervous system, the enteric nervous system, the neuroendocrine and neuroimmune systems, and the GM itself, which modulates neurotransmitter production, influences brain development and myelination patterns, and produces metabolites able to impact behavior in mice (6, 7). Indeed, the concept of the brain-gut axis (GBA) has been extended to the microbiota-gut-brain axis (MGBA) (8), to account for the complex networks existing between the GM and the CNS. Although previous studies reported a link between GM colonization and the CNS (9), recent evidence showed that dynamic microbiota-host interaction may alter brain-gut signaling and increase the risk of neurodevelopmental disorders. The concept of critical parallel-interacting windows in neurodevelopment and GM colonization provides new ways to develop novel therapeutic interventions modulating the microbiota to fight neurodevelopmental deficits and brain disorders.

Several studies have reported GM variation in children with neurological impairment (NI). Lee et al. (10) reported that children with intractable epilepsy (IE) had a significantly less variegate GM than healthy controls; Huang C et al. (11) described distinct GM composition and functions in children with cerebral palsy and epilepsy (CPE). These shreds of evidence suggest that GM provides a therapeutic target for the treatment of neurodevelopmental disorders.

On the other hand, feeding and diet, together with gastrointestinal disorders linked to neurological disability, may in themselves contribute to the immaturity and dysfunction of the GM, an imbalance in nutrient/energy intake, and, ultimately, malnutrition.

Nutritional management and treatment of gastrointestinal symptoms in children with NI have become a challenge in the past decades. In these children, malnutrition can occur frequently due to inappropriate dietary energy intake, oral motor dysfunction, increased nutrient losses, increased basal metabolic rate, and physical exertion, leading to the need for artificial nutritional support to ensure growth, counteract morbidity and mortality, and improve overall health and quality of life for both patients and their families (12–14). The European Society of Gastroenterology, Hepatology, and Nutrition (ESPGHAN) position papers clarified the criteria for the diagnosis of oropharyngeal dysfunction, the assessment, and monitoring of nutritional status, the standardization of nutritional requirements, and the gastrointestinal management of children with NI (14). The assessment of nutritional status and continuous monitoring are mandatory to prevent overfeeding or underfeeding (12–14). Nutritional support is normally provided enterally [enteral nutrition (EN)] rather than parenterally, assuming competency of the gastrointestinal tract (12–14).

Long-term EN (LTEN), especially when used exclusively, can deeply affect the GM taxonomic composition and functions. In general terms, LTEN implies a profound change in the provision of nutrients to the gut and this, together with metabolic stress and the use of drugs, normally administered to these patients, is responsible for a marked dysbiosis (15, 16).

A number of reports have addressed changes in GM composition following the intervention of exclusive enteral nutrition (EEN) as the first-line therapy in the setting of IBDs in children (17). Scarce data are available for the effects of EN in other pediatric settings (18). For example, examinations of intestinal microbial diversity were conducted in children with NI performing blenderized enteral nutrition (BEN). A significant improvement in gut bacterial diversity following BEN was reported in a sample of twenty children with rapidly progressive degenerative disorders. The reduction of *Proteobacteria* in the feces showed the benefit of BEN in preserving a healthy gut microbiota (19). Although increased gut microbial diversity may improve gastrointestinal mobility, the use of BEN still raises concerns and is not currently the first choice (13, 14, 20).

In this study, using amplicon sequencing, we investigated the fecal microbiota composition in a pediatric population suffering from severe NI and under exclusive LTEN and compared it to sex- and age-matched controls. A correlation between GM and metabolic profile, focusing on insulin resistance and glycemic derangement, was also considered.

## Patients And Methods

### Patients

We retrospectively enrolled 30 malnourished (BMI-z score ≤ 2) patients (16 male children/14 female children, aged 2–18 years) with severe neurological impairment [level 5 according to the Gross Motor Function Classification System (21)], who were bedridden and lived at home or in sheltered communities. Diagnoses included cerebral palsy due to hypoxic-ischemic damage (36.6%), severe psychomotor delay in dysmorphic syndromes (30%), and epileptic encephalopathy (33.4%). The patients were referred for auxological evaluation or the management of nutritional support. All of them were fed through EN by tube. Clinical and auxologic parameters, as well as metabolic and endocrinological assessments, were recorded for all patients.

In addition, 21 patients (11 male children/10 female children) with normal weight, healthy subjects, comparable for age and sex, referred by their general practitioner or primary care pediatrician for auxological evaluation, were included as controls for the fecal microbiota evaluation.

The study was approved by the Institutional Review Board (ARNAS Civico-Di Cristina-Benfratelli, PA register n. 354 Civico 2016) according to the 1964 Declaration of Helsinki. After receiving information on the nature of the study, written informed consent was obtained from the parents/guardians of the participating children.

### Methods

#### Auxological Parameters

Physical examination of the patients included: evaluation of weight, height, and body segment lengths according to Stevenson's method (22), waist circumference (WC), body mass index (BMI), and BMI *z*-score, and evaluation of pubertal stage according to Marshall and Tanner (23, 24). These parameters were determined to assess nutritional status and growth.

Blood pressure (BP) measurements were also performed as previously detailed (25). Increased systolic or diastolic BP (respectively: SBP and DBP) were defined as values exceeding the 95th percentile for age and sex (26).

Based on gestational age and birth weight, children were defined as appropriate (birth weight ≥ 10th percentile) and small for gestational age (birth weight < 10th percentile) (27).

#### Biochemical Parameters

Blood samples were drawn in the morning, after overnight fasting. Metabolic and hormonal blood assays included: fasting blood glucose (FBG), insulin, total cholesterol, high-density lipoprotein (HDL) cholesterol, triglycerides (TGs), Glutamic Oxaloacetic Transaminase (GOT), Glutamate Pyruvate Transaminase (GPT), and Gamma-glutamyl transpeptidase (GGT) (25).

Values exceeding the 95th percentile for age and sex for TG, and below the 5th percentile for HDL cholesterol were considered representative of impairment in lipid fasting levels (28).

Insulin resistance was determined by means of the homeostasis model assessment for insulin resistance (HOMA-IR) using the following formula: insulin resistance = (insulin × glucose)/22.5 (29). Impaired insulin sensitivity (ISI) was defined with HOMA-IR that exceeded the 97.5th percentile for age, sex, and pubertal stage (30).

#### Amplicon Sequencing (V3–V4 Regions of 16SrRNA)

Fecal samples collected from patients and sex- and age-matched controls were kept at −80°C till analysis. The use of biotics before collecting the fecal stool was excluded in all patients and controls. DNA was extracted from 200 mg of each sample by the QIAamp Fast DNA Stool Mini Kit (Qiagen; Hilden, DE), following the manufacturer's protocol. The DNA concentration of each sample was assessed fluorometrically. For amplicon production, the V3–V4 hypervariable regions of the prokaryotic 16S ribosomal RNA (rRNA) gene was targeted (31). PCR was set up in a 50-μl volume with template DNA, 1x HiFi HotStart Ready Mix (Kapa Biosystems, Wilmington, MA), and 0.5 μM of each primer. The amplification was carried out on a Bio-Rad T100 thermal cycler (Bio-Rad, Hercules, CA) and included: initial denaturation (95°C for 3 min); 30 cycles at 94°C for 30 s (s), 55°C for 30 s, 72°C for 30 s; and final extension (72°C for 5 min). Clean-up of amplicons was performed using Agencourt AMPure XP SPRI magnetic beads (ThermoFisher Scientific). Illumina sequencing libraries were finally prepared through the link of indexes (Nextera XT Index Kit, Illumina, San Diego, CA), quantified using a Qubit 3 Fluorometer (ThermoFisher Scientific, Waltham, MA), normalized, and pooled. Libraries were subjected to paired-end sequencing (2 x 300 bp format) on an Illumina MiSeq platform at BMR Genomics (Padova, Italy).

#### Bioinformatics and Community Analyses

The bioinformatics analysis of sequencing data was based on the Mothur pipeline (32). Briefly, raw FASTQ files were quality-filtered using Trimmomatic (33), and high-quality reads were analyzed following the SOP Mothur procedure. Chimeric sequences were identified and removed using UCHIME (34). The remaining sequences were clustered into Operational Taxonomic Units (OTUs) at the 97% homology level using VSEARCH (35). OTUs were finally annotated, and taxonomy assigned against the reference database SILVA (36). The main ecological indexes of within-sample, α, diversity (Shannon, Chao, inverse Simpson, and Observed Richness) were computed using Mothur. Diversity in composition among samples (β-diversity) was evaluated at all taxonomic ranks by plotting the relative heatmap using the function heatmap.2 of the Gplots R library (37), and the relative principal coordinates analysis (PCoA) using the R library Ade4 (38) and Permanova analysis using the R library Vegan (39). Microbial profiles of patients and controls were compared for evidence of statistically significant differences in bacterial composition in taxa abundance. Comparisons were performed using the Wilcoxon–Mann–Whitney test with a significance threshold (*p*-value) set to 0.05.

## Results

### Demographic, Growth, Clinical, and Metabolic Features of Patients With NI

Table 1 lists the relevant demographic and clinical features of these patients. Their mean age is 12.1 ± 5.9 years, without significant deviations in the sex ratio. The pubertal stage is Tanner 1 in 9 cases (30%), Tanner stage 2–3 in 4 (13.3%), and Tanner stage 4–5 in 17 (56.6%). Birthweight was recorded in 18/30 (60%) of cases and was appropriate in 16/18 (88.9%).

**Table 1:** Demographic, growth, and clinical features of the enrolled patients.

**NI patients**	
Male:female ratio	16:14
Mean age in years (±standard deviation)	12.1+/−5.9
Pubertal stages	
0 (Tanner stage 1)	
1 (Tanner stage 2–3)	
2 (Tanner stage 4–5)	9 4 17
Nutritional support	
Enteral continuous	
Enteral bolus	11 19
BMI-SDS (kg/m^2^ ±standard deviation)	−1.61 ± 2.50
Waist circumference (cm ±standard deviation)	71.07 ± 15.12
Waist-to-height ratio (± standard deviation)	0.53 ± 0.11
Use of anticonvulsive drugs	28/30
Use of antibiotics (last 3 months)	3/30
Use of others medicaments	30/30

*BMI-SDS,standardized BMI*.

All patients are fed through an exclusive LTEN regimen, continuous in 11/30 subjects (36.7%) and bolus in 19/30 (63.3%). All patients are fed with a whole-protein-based enteral formula with a similar energy value and composition. Specifically, no fiber and/or biotics supplementation in enteral formulas was present.

Finally, Table 1 lists the pharmacological therapies administered to NI subjects. Of 30, 28 (93.3%) of them assume anticonvulsive drugs (at least two of the following: phenobarbital, valproic acid, phenytoin, lamotrigine, topiramate, carbamazepine, and clonazepam). Furthermore, 3/30 subjects (1%) have assumed antibiotics in the past 3 months before sampling. All patients are given other drugs, e.g., antihypertensive drugs, antiacids, and hormones, such as L-thyroxine.

Metabolic parameters are shown in Table 2. Of 30 subjects, 24 (80%) of the NI subjects display at least one altered parameter. An impaired insulin sensitivity (ISI) is evident in 50% of the patients, one of which diagnosed with type 2 diabetes. Impairments in TGs, total cholesterol, and HDL-cholesterol characterize 36.7, 1, and 16.7% of the cohort, respectively. Finally, 23.3% of the cases were present with hypertension and 26.6% with increased transaminase levels.

**Table 2:** Metabolic parameters of patients with neurological impairment (NI).

**Parameters**	**Values**
Fasting blood glucose (mg/dl) -Pathological values	82.0 ± 54.4 1/30
Insulin (μU/ml) -Pathological values	22.2 ± 21.5 15/30
HOMA-IR -Pathological values	4.5 ± 4.7 15/30
Triglycerides (mg/dl) -Pathological values	116.4 ± 71.1 11/30
Total Cholesterol (mg/dl) -Pathological values	146.8 ± 36.5 3/30
HDL-Cholesterol (mg/dl) -Pathological values	45.6 ± 14.7 5/30
Diastolic pressure (mmHg) Systolic pressure (mmHg) -Pathological values	67.7 ± 12.5 108.2 ± 15.7 7/30
GOT (mU/ml) GPT (mU/ml) GGT (mU/ml) -Pathological values	27.8 ± 13.9 21.6 ± 19.8 30.0 ± 19.8 8/30

*GOT, Glutamic Oxaloacetic Transaminase; GPT, Glutamate Pyruvate Transaminase; GGT, Gamma-glutamyl transpeptidase; HOMA-IR, homeostasis model assessment for insulin resistance*.

### Taxonomic Structure and Ecological Parameters of Fecal Bacterial Communities in NI Children Under LTEN

To investigate the composition of fecal bacterial communities in NI children under LTEN, and compare it with that of controls (CTRL) matched for sex and age, we produced and sequenced 50 amplicons (29 from patients with NI and 21 from CTRL), comprising the V3–V4 regions of 16S rRNA gene, as detailed in Materials and methods. A total of 3.9 million reads were obtained and clustered into 4,545 OTUs at 97% homology level. After the application of low count and low variance filters, OTUs were representative of 22 bacterial phyla, 40 classes, 80 orders, 160 families, and 382 genera.

**Figure 1:**
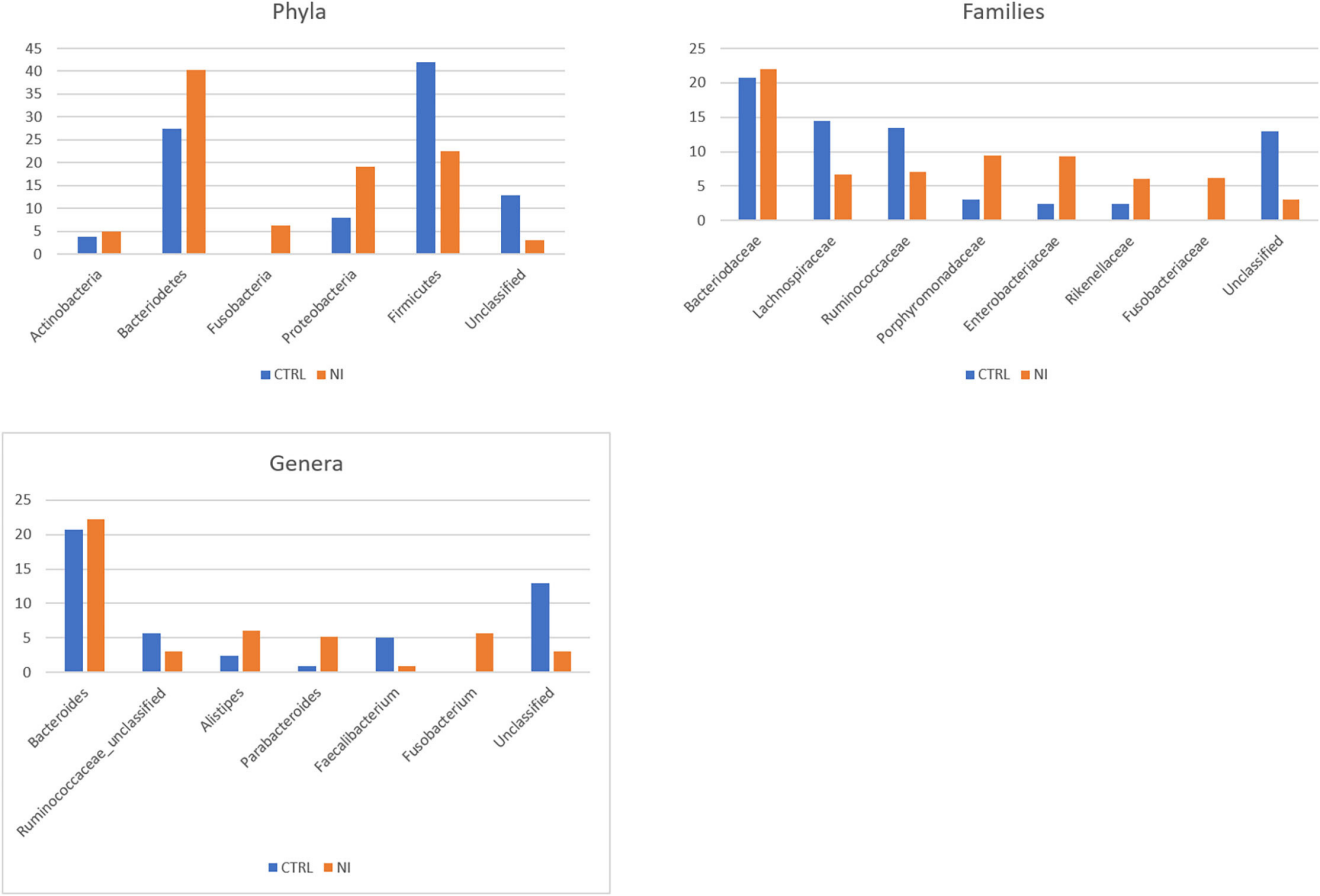
Taxonomic composition of the gut microbiota in patients with neurologically impairment (NI) compared with sex- and age-matched healthy controls (CTRL). Average relative abundance of the most represented phyla, families and genera identified in the two groups. Only taxa whose relative abundance is > 5% in at least one group are included.

The average relative abundance for the most represented phyla, families, and genera in NI and CTRL groups is shown in Figure 1. As for phyla, *Firmicutes* and *Bacteriodetes* are the most abundant and, together with *Proteobacteria*, account for most of the bacterial diversity. *Proteobacteria* increased in patients with NI (19.1%) as compared with controls (7.9%). Patients with NI also appear to be characterized by the expansion of the phylum *Fusobacteria* (6.2%), virtually absent in controls, and by the reduction of *Firmicutes* (22.6 vs. 42% in controls). Concerning the taxonomic rank of families, the most abundant in both groups is *Bacteriodaceae* (20.7% in CTRL and 22% in NI). Patients with NI present a reduction in the *Clostridiales* families of *Lachnospiraceae* (6.7 vs. 14.5% in CTRL) and *Ruminococcaceae* (7.1 vs. 13.5% in controls). On the other hand, they are characterized by an increase in the *Bacteriodales* families of *Porphyromonadaceae* (9.5 vs. 3% in controls) and *Rikenellaceae* (6 vs. 2.4%). *Enterobacteriaceae* (order: *Enterobacterales*) account for 9.3% of the bacterial families in patients (vs. 2.4% in CTRL), and *Fusobacteriaceae*, (order: *Fusobacteriales*), nearly undetectable in controls, reached 6.2% in the NI cohort. As for genera, the picture largely reflects what is seen for families, with patients with NI characterized by an expansion of *Alistipes* (family: *Rikenellaceae*, 6 vs. 2.4% in CTRL) and *Fusobacterium*, which reaches 5.7% in patients and is virtually undetectable in controls. *Parabacteriodes* (family: *Tannerellaceae*) appears to increase as well (6 vs. 2.4%). On the other hand, *Faecalibacterium*, belonging to the *Ruminococcaceae* family, appears strongly decreased in patients (0.9 vs. 5.1% in controls).

The comparison of within-sample diversity (α-diversity) indexes evidenced a trend of decreasing values in patients that, however, did not reach statistical significance (not shown). To evaluate how bacterial taxa were differentially distributed in the NI and CTRL groups, differences in composition among samples (β-diversity) were computed as detailed in the Materials and methods. A clear separation was observed between the two groups at all taxonomic rankings, which was verified using the PERMANOVA test (*p*-value < 0.001 for all taxonomic levels).

**Figure 2:**
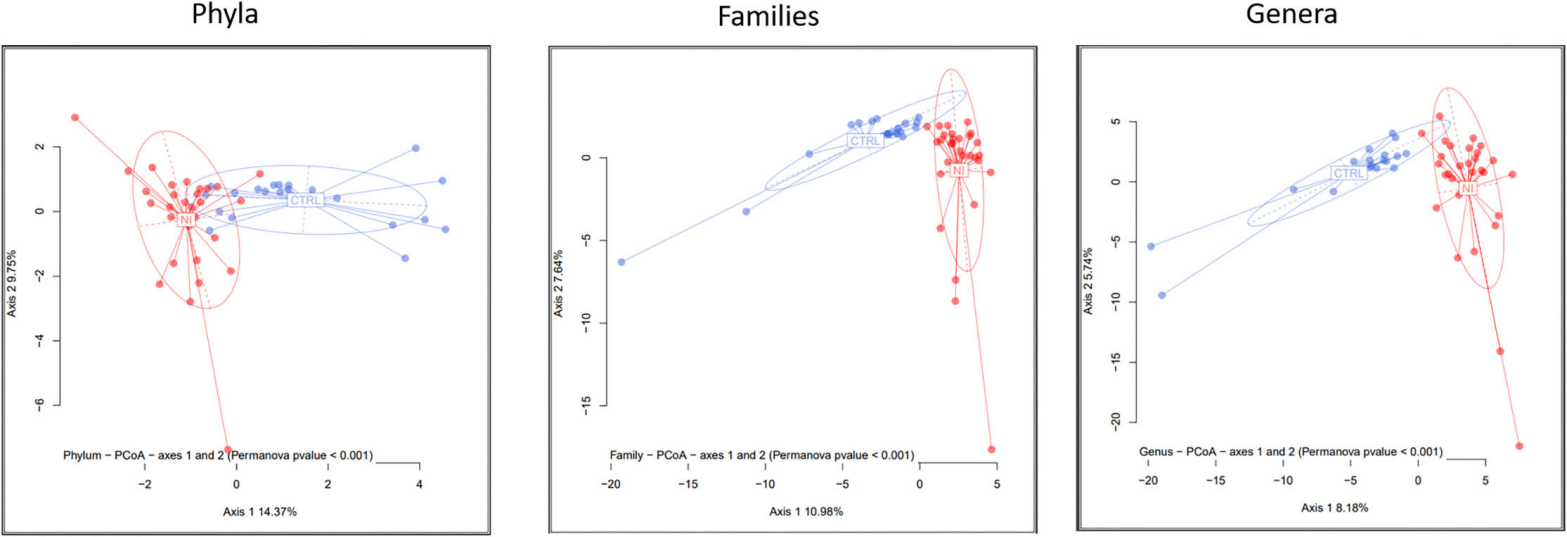
A β-diversity analysis. The microbiota distances were visualized through Principal Coordinates Analysis (PCoA). The figure shows the taxonomic levels of phyla, families, and genera. The significance threshold (*p*-value) for the PERMANOVA was set < 0.001. NI, children suffering from a neurological impairment, subjected to long-term enteral nutrition; CTRL, controls matched for sex and age.

Figure 2 shows the principal coordinates analysis (PCoA) for the taxonomic levels of phyla, families, and genera. The differential clustering appeared dictated solely by the NI and LTEN conditions vs. controls: indeed, when dividing NI children into 2 sub-categories, according to insulin resistance or sensitivity (as shown in Materials and methods), no significant differences in bacterial profiles and β-diversity plots were evidenced between these two groups of patients (not shown).

To better define differential abundance and identify which taxa contributed to the clear separation between patients with NI and controls observed in the β-diversity analyses, Wilcoxon–Mann–Whitney and Kruskal–Wallis rank sum non-parametric tests were applied, using a significance threshold (*p*-value) set to 0.05. Table 3 lists the taxa displaying significantly different relative abundances in NI and CTRL groups. Among taxa significantly enriched in NI children: the genus *Fusobacterium*, virtually absent in controls, and its higher taxonomic rankings till the phylum *Fusobacteria*; the phylum *Proteobacteria* and, within it, the orders *Burkholderiales* and *Enterobacteriales*. Within the latter, the family *Enterobacteriaceae* and the genus *Klebsiella*. *Parabacteroides* is another genus strongly expanded in patients and virtually absent from controls, together with *Cloacibacillus*. Concerning the latter, all its corresponding higher taxonomic rankings are detectable only in patients, till the phylum *Synergistia*. Two families belonging to the phylum *Bacteroidetes* (order: *Bacteriodales*), finally, display a robust increase in patients: *Porphyromonadaceae* and *Rikenellaceae*. Other taxa, on the contrary, appear strongly depleted in NI children: the phylum *Firmicutes* and, within it, the class *Clostridia* and its order *Clostridiales*. The strong under-representation of the *Clostridiales* families *Lachnospiraceae* and *Ruminococcaceae*, and, within the latter, of the genera *Gemmiger* and *Faecalibacterium*, complete the picture for the Firmicutes phylum. Finally, *Bifidobacterium* (together with *Bifodobacteriaceae* and *Bifidobacteriales*) also appears significantly decreased in patients compared with controls.

**Table 3:** Taxa displaying significantly different relative abundances in NI and CTRL groups.

**Taxon**	**Mean relative abundance**		***p*-value**
	**CTRL**	**NI**	
**Phyla**			
*Fusobacteria*	0.002	6.2	2 x 10^−9^
*Proteobacteria*	7.9	19.1	1.3 x 10^−5^
*Synergistetes*	0.1	2.3	2 x 10^−5^
*Firmicutes*	42	22.6	8.3 x 10^−6^
**Classes**			
Phylum: *Fusobacteria*			
*Fusobacteriia*	0.002	6.2	2 x 10^−9^
Phylum: *Proteobacteria*			
*Betaproteobacteria*	0.83	5.4	6.8 x 10^−8^
Phylum: *Synergistetes*			
*Synergistia*	0.1	2.3	2 x 10^−5^
Phylum: *Firmicutes*			
*Clostridia*	34.6	14.5	7.5 x 10^−9^
**Orders**			
Phylum: *Actinobacteria*			
*Bifidobacteriales* (class: *Actinobacteriia*)	3.4	1	1.2 x 10^−4^
*Coriobacteriales (*class*: Coriobacteriia)*	0.34	3.5	3.5 x 10^−7^
Phylum: *Fusobacteria*			
*Fusobacteriales (*class*: Fusobacteriia)*	0.002	6.2	2 x 10^−9^
Phylum: *Proteobacteria*			
*Burkholderiales* (class*: Betaproteobacteria)*	0.8	5.4	6.8x10^−8^
*Enterobacteriales* (class*: Gammaproteobacteria)*	2.4	9.3	5.8 x 10^−4^
Phylum: *Synergistetes*			
*Synergistales* (class*: Synergistia)*	0.1	2.3	2 x 10^−5^
Phylum: *Firmicutes*			
*Clostridiales* (class*: Clostridia)*	34.1	14.5	8.9 x 10^−9^
**Families**			
Phylum: *Actinobacteria*			
*Bifidobacteriaceae* (order: *Bifidobacteriales*)	3.4	1	1.2 x 10^−4^
*Coriobacteriaceae* (order: *Coriobacteriales*)	0.3	3.5	3.5 x 10^−7^
Phylum: *Bacteroidetes*			
*Porphyromonadaceae* (order: *Bacteriodales*)	3	9.5	2 x 10^−6^
*Rikenellaceae* (order: *Bacteriodales*)	2.4	6	3.8 x 10^−4^
Phylum: *Fusobacteria*			
*Fusobacteriaceae* (order: *Fusobacteriales)*	0.002	6.2	2.6 x 10^−9^
Phylum: *Proteobacteria*			
*Enterobacteriaceae* (order: *Enterobacteriales*)	2.4	9.3	5.8 x 10^−4^
Phylum: *Synergistetes*			
*Synergistaceae* (order*: Synergistales)*	0.1	2.3	2 x 10^−5^
Phylum: *Firmicutes*			
*Lachnospiraceae* (order*: Clostridiales)*	14.5	6.7	1.7 x 10^−3^
*Ruminococcaceae* (order*: Clostridiales)*	13.5	7.1	5.6 x 10^−5^
**Genera**			
Phylum: *Actinobacteria*			
*Bifidobacterium* (family: *Bifidobacteriaceae)*	3.4	1	1.2 x 10^−4^
Phylum: *Bacteroidetes*			
*Parabacteriodes* (family: *Tannerellaceae)*	0.9	5.2	1.5 x 10^−7^
Phylum: *Fusobacteria*			
*Fusobacterium* (family: *Fusobacteriaceae)*	0.002	6.2	3.4 x 10^−9^
Phylum: *Proteobacteria*			
*Klebsiella* (family: *Enterobacteriaceae)*	0.6	3.4	2 x 10^−7^
Phylum: *Synergistetes*			
*Cloacibacillus* (family: *Synergistaceae)*	/	2.3	4.1 x 10^−9^
Phylum: *Firmicutes*			
*Gemmiger* (family: *Ruminococcaceae)*	4	0.04	7.9 x 10^−9^
*Faecalibacterium* (family: *Ruminococcaceae)*	5.1	0.9	4.6 x 10^−7^

*Wilcoxon–Mann–Whitney and Kruskal–Wallis rank sum non-parametric tests were applied, using a significance threshold (p-value) set to 0.05. For each taxon, the immediately higher taxonomic ranking is indicated, together with the phylum*.

## Discussion

Inadequate nutritional status and disability are intimately linked (40). Malnutrition can occur in children with NI as a result of imbalance in nutrient/energy intake, oral motor dysfunction, increased loss of nutrients and basal metabolic rate, and physical inactivity (25, 41), necessitating long-term dependence on EN for providing the entire (or partial) caloric intake (15, 16, 25).

A 2021 ESPGHAN position paper aimed to standardize the nutritional and gastrointestinal management of children with NI (14). Initiation of EN is recommended in cases of inefficient, unsafe, and/or stressful oral energy and fluid intake, and gastrostomy placement is considered the primary way to provide enteral access. A standard EN with a standard polymeric formula (1 kcal/ml) suitable for age is a common choice. The ESPGHAN recommendation advises caution in the administration of home mixed diets due to concerns about their safety and nutritional adequacy to maintain an adequate nutritional status (14).

Diet is a strong modifier of the GM and its gene content, especially in children, whose gut bacterial communities are subject to rapid changes. Strong evidence links the microbial composition of the gut to situations of malnourishment in children, due to the pivotal role in extracting and metabolizing dietary ingredients (42, 43). Indeed, the composition and functional capacity of the gut microbiota appear to be altered in childhood undernutrition (44). In particular, an “undernourished microbiota” has been described as “immature” based on an altered diversity, a less efficient nutrient utilization, and strong compositional differences in specific bacterial taxa (e.g., enrichment in pathobionts and inflammogenic species, often aerotolerant and belonging to *Proteobacteria*), as compared with age-matched children exhibiting a consistently healthy growth (43, 45).

On the other hand, data on different GM compositions in children with neurological damage have been reported. Lee et al. (10) reported that children with intractable epilepsy (IE) had significantly greater diversity in GM than healthy controls. The species biomarkers for IE included the *Enterococcus faecium* group, the *Bifidobacterium longum* group, and *Eggerthella lenta*. Low levels of *Bacteroidetes* and high levels of *Actinobacteria* were also found in epileptic children compared with healthy controls. Huang et al. (11) published a case-control study showing significant differences in GM composition between children with cerebral palsy and epilepsy (CPE) as compared with a healthy group. In detail, significantly increased relative abundances in *Bifidobacterium, Streptococcus, Akkermansia, Enterococcus, Prevotella, Veillonella, Rothia*, and *Clostridium (IV*) and, on the other hand, significant reductions in *Bacteroides, Faecalibacterium, Blautia, Ruminococcus, Roseburia, Anaerostipes, and Parasutterella* characterized the CPE group.

In this study, we investigated, for the first time to the best of our knowledge, the consequences of LTEN in a neurologically impaired pediatric population, focusing on the GM.

In agreement with what was described above, the overall comparison of GM composition in NI children under LTEN and age- and sex-matched subjects revealed a clear clustering of the two groups, as a reflection of profound differences in the bacterial populations. In general, the differential taxonomic picture in NI children under LTEN seems to mirror a profound dysbiotic condition, in which protective taxa as Short-Chain Fatty Acids (SCFA) producers and anti-inflammatory taxa appear severely depleted (e.g., the *Clostridiales* families of *Lachnospiraceae* and *Ruminococcaceae*), while known pathobionts (*Gammaproteobacteria* and *Klebsiella*) or emerging pathogens (*Synergistales* and *Cloacibacillus, Fusobacterium*) are significantly enriched. In general terms, this picture is reminiscent of gut microbiota changes found in inflammatory disorders, as well as in malnourished children, as will be better detailed above.

Moving on to more details on the differential taxa, an interesting point is the strong depletion of the genus *Faecalibacterium* (family: *Ruminococcaceae*) whose only known species is *Faecalibacterium prausnitzii*. Normally, this is an abundant species, and one of the main butyrate producers, with protective and anti-inflammatory properties, and the capacity of enhancing the intestinal barrier function (46). Its abundance is reduced in different intestinal disorders (type 2 diabetes, colorectal cancer, and the two main IBDs: Crohn's disease (CD) and ulcerative colitis (UC), and its role in promoting gut health is increasingly recognized, to the point that it has been suggested to consider its monitoring as a biomarker to assist in gut diseases diagnostics and prognostics (46). Other SCFA producers belonging to *Firmicutes*, known to diminish in many inflammatory disorders among which pediatric IBD, appear depleted, e.g. *Lachnospiraceae* and *Gemmiger* (family: *Ruminococcaceae*), another genus of commensal bacteria linked to a healthy gut status (47). Concerning *Proteobacteria*, many concordant pieces of evidence link an unstable gut microbiota community enriched in this phylum (rich in aerotolerant and pro-inflammatory taxa, e.g., those belonging to *Enterobacteriaceae*) to a plethora of infective and inflammatory conditions, and undernutrition (48, 49). Their outgrowth is generally recognized as a sign of microbial dysbiosis in the gut (49). Another interesting point is the enrichment in the NI microbiota of emerging pathogens: (i) *Cloacibacillus*, belonging to the *Synergistetes* phylum (50) and considered an intestinal opportunistic pathogen; (ii) *Fusobacterium*, able to strongly activate host inflammatory responses and linked to several diseases, as well as a promoter of colorectal carcinogenesis (51).

The dysbiotic picture that emerges for these taxa is strongly reminiscent of what is repeatedly reported in the literature for children malnourished as a consequence of inadequate food intake, often coming from low-income Western African and Asian countries (42, 43): the strong depletion of several gut obligate anaerobes belonging to *Firmicutes, Bacteriodetes*, and *Actinobacteria*; and the enrichment in aerotolerant taxa, many of which *Proteobacteria*, more competitive and starvation resistant, and able to metabolize broader classes of substrates, such as amino acids (44, 45, 52). As for the link to our results, it is to be noted that the depletion of *Faecalibacterium* was found to be the most highly discriminatory bacterial taxonomic biomarker to distinguish malnourished children from age-matched controls exhibiting healthy growth (42). The “anaerobic depletion” also encompasses *Bifidobacterium*, whose reduction has been described as one of the first steps in gut microbiota alterations associated with severe acute malnutrition (45).

It is interesting to underline that, while most of these data come from pediatric populations in low-income countries, we retrieve comparable taxonomic biomarkers and dysbiosis pictures in a completely different context, regardless of geographic provenience, lifestyle, and therapeutic assessment.

Another important point is that data on the effects of EN on gut microbiota are scarce, and, at least for pediatric populations, generally limited to the use of exclusive EN as the first-line therapy in children neo-diagnosed with Crohn's disease and not always consistent among studies (53). Our article adds new results for a completely different pediatric setting.

Finally, it is interesting to cite a previous work investigating the effects of parenteral nutrition (PN) on gut microbiota structure (52). The authors retrieved similar changes in community structure, in terms of decreased relative abundance of *Firmicutes* and increased percentages of *Bacteroidetes* and *Proteobacteria*.

We are aware that our study has some limitations. First of all, a relatively small sample size could limit the power of the analysis, even if the differential clustering and bacterial taxonomic pictures of the two groups are supported by strong statistical evidence. Second, we considered a cohort of children with a severe grade of disability that could in itself contribute to the severity of dysbiosis; a comparison with a group of children with moderate disabilities would be useful in future studies. Finally, long-term anticonvulsant drugs may be associated with GM alterations. However, all the subjects were on anticonvulsant medications and this factor can be considered as a systematic error. Despite these limitations, the present study presents a first picture of the relevant modifications in GM taxonomic composition found in NI children and adolescents under LTEN. This could represent the premise of a broader process for improving the management of these children, as it implicitly suggests possible routes and targets to try to alleviate the observed dysbiosis, and improve the overall clinical conditions, *in primis*, the gastroenterological problems, which are often considerable (54). A relevant example could be *F. prausnitzii*, one of the most heavily depleted taxa, whose use as a probiotic species is currently under evaluation. Other two strategies that could potentially be explored are the supplementation with prebiotics able to enhance populations of SCFA producers, with a special focus on the anti-inflammatory butyrate, of which *F. prausnitzii* is a major producer, or giving the postbiotic butyrate directly.

Finally, further research is desirable, especially to establish whether dysbiosis may be a primary manifestation of a secondary consequence of chronic impaired health and malnutrition.

## Conclusion

Our results suggest that LTEN can influence the GM taxonomic composition in neurologically impaired children. However, the role of bidirectional interaction between GI impairment/immaturity and the CNS in disabled children probably remains a key player in dysbiosis. Additional factors, such as drugs and physical inactivity, could not be excluded.

The physiologic, metabolic, and immunologic consequences of changes in GM and how they might contribute to associated morbidities and sequelae of malnutrition still need to be fully elucidated. New insights are presented here and addressed in patients that are not usually investigated in this sense.

Future interventions based on dietary modulations of the GM, e.g., through biotics-based interventions, to support a healthy microbiota in these children, should be investigated as a tool to help to combat their malnourishment and improve their general conditions, clinical outcomes, and quality of life, even if keeping in mind that restoration of health status may be incomplete. In this regard, longitudinal studies would certainly be useful to clarify the long-term efficacy of nutritional interventions. Additionally, interventional studies before and after a planned EN will be useful to define the specific role of EN on dysbiosis.

## Data Availability Statement

The data presented in this study have been deposited in the NCBI BioSample database, BioProject ID: PRJNA842554.

## Ethics Statement

The studies involving human participants were reviewed and approved by ARNAS Civico-Di Cristina-Benfratelli, PA Register No. 354 Civico 2016. Written informed consent to participate in this study was provided by the participants' legal guardian/next of kin.

## Author Contributions

SP and VC participated in the study design and project management. SP, VC, EV, FC, GP, EB, CB, and GZ were responsible for the conceptualization and design of forms, data management and quality control, and writing and editing the manuscript. SP, FC, and CB were responsible for the statistical analysis and documentation. VC, GP, and EB were responsible for the recruitment of participants. SP, VC, EV, GP, CB, and GZ participated in the study supervision. All authors contributed to the article and approved the submitted version.

## Conflict of Interest

The authors declare that the research was conducted in the absence of any commercial or financial relationships that could be construed as a potential conflict of interest.

## Publisher's Note

All claims expressed in this article are solely those of the authors and do not necessarily represent those of their affiliated organizations, or those of the publisher, the editors and the reviewers. Any product that may be evaluated in this article, or claim that may be made by its manufacturer, is not guaranteed or endorsed by the publisher.
